# Factors related to HIV testing frequency in MSM based on the 2011–2018 survey in Tianjin, China: a hint for risk reduction strategy

**DOI:** 10.1186/s12889-021-11948-6

**Published:** 2021-10-20

**Authors:** Zhongquan Liu, Yang Chen, Tingting Yao, Tiantian Zhang, Desheng Song, Yuanyuan Liu, Maohe Yu, Jie Xu, Zhijun Li, Jie Yang, Zhuang Cui, Changping Li, Jun Ma

**Affiliations:** 1 STD & AIDS Control and Prevention Section, Tianjin Center for Disease Control and Prevention, Tianjin, China; 2 The Second People’s Hospital of Guiyang, Guiyang, Guizhou China; 3 Department of Epidemiology and Health Statistics, School of Public Health, Tianjin Medical University, Tianjin, China; 4 National Center for AIDS/STD Control and Prevention, Beijing, China; 5 GAP Program Office of US CDC, Atlanta, GA USA; 6 Tianjin Shenlan Public Health Counseling Service Center, Tianjin, China

**Keywords:** HIV testing, HIV, Men who have sex with men, HIV testing frequency

## Abstract

**Background:**

In recent years, HIV testing has become one of the effective strategies to reduce the risk of the infection. Frequent quarterly HIV testing can be cost effective. Therefore, an in-depth study of factors related to the testing behavior of men who have sex with men (MSM) were analyzed to optimize intervention strategies.

**Methods:**

From March 2011 to October 2018, the project was implemented in a Tianjin (China) bathhouse, and 5165 MSM were surveyed using snowball sampling. Factors related to HIV testing behavior were analyzed by ordinal logistic regression analysis after grouping according to testing frequency, and comprehensive analysis was performed.

**Results:**

The multivariate logistic analysis showed that 6 variables including young MSM (OR = 0.67, 95% CI: 0.49–0.92, *p* = 0.01), low-educated MSM (OR = 0.60, 95% CI: 0.48–0.77, *p* < 0.0001), low HIV/AIDS knowledge (95% CI: 0.57–0.83, *p* < 0.0001), marital status (OR = 1.30, 95% CI: 1.07–1.57, *p* = 0.007), acceptance of condom promotion and distribution (OR = 14.52, 95% CI: 12.04–17.51, *p <* 0.0001), and frequency of condom use (*p* < 0.05) could link to HIV testing behaviors.

**Conclusions:**

In order to achieve the 95–95-95 goal, target publicity, HIV/AIDS education and promotion of HIV self-testing kits should be carried out to encourage frequent HIV testing among MSM who are young (especially students), married to women, poorly educated and who are reluctant to always use condoms.

**Supplementary Information:**

The online version contains supplementary material available at 10.1186/s12889-021-11948-6.

## Background

Men who have sex with men (MSM) have been the focus of HIV prevention in high-risk groups. According to the 2019 UNAIDS report [1], the proportion of new HIV infections amongst MSM is increasing annually. The epidemiological evidence also suggests that the greatest priority in China’s response to HIV is prevention programs targeting MSM [2]. Owing to a lack of widespread HIV testing, the majority of the infected population is not on effective antiretroviral therapy [3]. In addition, HIV testing is a key strategic tool for HIV prevention in China. It has been reported in the literature that more frequent HIV testing among MSM can reduce expenditure in the health sector [4]. Studies generally focus on comparing HIV testing strategies to analyse cost effectiveness but not on actual frequency of testing among MSM [5]. The popularization of instant messaging technologies such as WeChat and Blue D in China have gradually changed some sexual behavior of MSM but the risk of HIV transmission has also increased [6]. Although HIV testing frequency has been investigated [7], the factors related to testing frequency have not been well described in conjunction with the behavioral factors in MSM. The Joint United Nations Programme on HIV/AIDS (UNAIDS) has recommended an ambitious 95–95-95 target to end the AIDS epidemic by 2030. It states that by 2030 at least 95% of PLHIV should know their status, 95% of people diagnosed with HIV should receive sustained ART and 95% of those on ART should achieve viral suppression [8]. The Center for Disease Control (CDC, USA) has recommended that high-risk groups should be tested for HIV at least once a year [9]. However, several studies have shown that HIV testing in MSM is not as satisfactory as expected [10, 11]. In 2016, the rate of HIV testing among high-risk groups in Europe was very low, and it was estimated that 25% of people living with HIV were undiagnosed [12]. In an MSM population survey in 20 cities in the United States, more than 35% of the population did not have HIV testing within the last 1 year as recommended by the CDC [13]. The mandatory surveillance system for Sexually Transmitted Infections (STIs) in England showed that of the 37,702 HIV-negative MSM in 2013–2014, 64% were tested for HIV within the last 1 year, and only 1% were tested every quarter [14]. In China, the proportion of undetected MSM population was as high as 57.4% [15]. Furthermore, less than 20% of MSM had been tested in the past year in Jinan, China [16]. There appears to be a paucity of literature investigating the factors which influence MSM testing for HIV amongst those who have not been tested for HIV in in the last one or more years. Many people have understood the importance of HIV testing especially after decades of HIV/AIDS-related knowledge publicity. However, there still appears to be a low testing rate in MSM who also appear not to have regular testing. Therefore, the factors which influence HIV testing in MSM needs further investigation especially to implement appropriate strategies to promote regular testing in MSM.

Our study explores a range of factors which may influence HIV testing in MSM. An analysis of the demographic, sexual behavior, HIV/AIDS related knowledge and HIV testing may provide insights for recommendations to improve the testing strategy in MSM in China.

## Methods

### Study population

The project was undertaken in a gay bathhouse in Tianjin (China) from March 2011 to October 2018. This bathhouse is frequented by a large and stable source of visitors bringing together many MSM from various parts of China. We collaborated with a private MSM organization called Shenlan which used professionally trained MSM (Investigators) to conduct the surveys and provide HIV/AIDS education.

### Study design

We used a prospective design involving snowball sampling since MSM in China are a marginalized and stigmatized group which can be difficult to reach. The recruited study participants notified their contacts, and those who volunteered to participate went to the bathhouse or the Shenlan Organization office to be recruited into the study.

### Survey questionnaire design

The survey was an electronic self-administered questionnaire which comprised demographic information, level of HIV/AIDS related knowledge, sexual behavior, drug use, STI status, self- awareness of HIV status, condom promotion, sex work, previous HIV testing behaviors (including within the last 1 year, more than 1 year, never tested) and HIV testing results.

Demographic and personal information included age, marital status, household registration, ethnicity, length of stay in Tianjin, education, frequency of attendance to bathhouses, and other places for seeking sexual partners.

Out of the eight HIV/AIDS knowledge related questions, more than six correct answers were considered as being a high level of HIV/AIDS knowledge. Sexual behavior questions included whether anal sex, group sex, commercial sex, heterosexual behavior had occurred in the past 6 months and whether condoms were used in these situations.

(The survey questionnaire can be found in the Supplementary Material). The questionnaire was developed for this study, and there was a version for young MSM [17].

### Study protocol

Participants were men who claimed to be MSM or had sex with men within the last 1 year. After the participants signed the informed consent, investigators conducted a one-to-one survey and also provided HIV/AIDS health education in a specially established private compartment. Investigators collected participants’ fingerprints to create an electronic file, record the participant study number and survey date. Participants were given access to tablet computers to complete the self-administered electronic questionnaires. The investigators communicated with the participants by using an MSM lingo which is a style of language used by this particular group of people in China thus promoting mutual trust to obtain reliable responses. After the questionnaire was completed, the participants were tested for HIV using blood (ACON, Hangzhou, Zhejiang province, China) or saliva (AWARE, Beijing, China) by using a rapid HIV test. While waiting for the results, the investigators checked the completed questionnaire to conduct a risk assessment of the participant and provided targeted HIV/AIDS health education and behavior recommendations. Positive and discrepant rapid tests were further confirmed by a Western Blot and a Nucleic Acid Test. Test results were communicated to participants in about a week. All participants who tested positive were accompanied by volunteer staff for treatment and medication.

### Ethics approval

This study was reviewed and approved by the Institutional Review Board of the National Center for AIDS/STD Control and Prevention, China CDC [IRB approve number: X130205267]. All participants signed an informed consent form before the survey started.

### Data analysis

Descriptive statistics were used to summarize the baseline characteristics of the study participants including demographic and sexual behavior information, HIV testing experience and results, HIV/AIDS related knowledge, condom promotion, etc. We applied ordinal logistic regression to analyze the association between the above factors and the frequency of HIV testing (first-tested, not annually tested, and annually tested) for MSM to find out who were more inclined to undergo annual HIV testing. The regression model used the backward method, with *p* = 0.05 as criteria for entering the model. The odds ratio (OR) and 95% confidence interval (CI) were calculated. All analyses were performed using SAS, v.9.4 (SAS Institute, Cary, NC, USA).

## Results

### Demographic characteristics of participants

Of the 5165 participants, 4316 MSM were included in the analysis while 849 were excluded due to unclear testing behavior. (Table 1). Among 4316 MSM, 1297 were first-time testers, 410 were non-annual testers and 2609 were annually-tested. Most of the MSM were over 24 years old (69.3%), had no partner (54.7%), non-Tianjin household registration (97.0%), and about 58.7% of MSM had lived in Tianjin for more than 2 years. The proportion of MSM with high school education was 37.9% while those with university education was 32%. The majority (60.5%) of MSM sought sexul partners from bathhouses, 32.0% used the internet and the rest used places such as parks and bars. Among the MSM surveyed, there were 216 (5.0%) male sex workers (MSW), with the highest proportion (6.5%) among annual testers. (Table 1).

**Table 1:** Demographic characteristics of different testers in MSM attending bathhouse

variable	total	First-tested	Non-annual	Annually-tested
		N (%)	N (%)	N (%)
Age				
< 24	1327 (30.7)	116 (8.9)	108 (26.3)	1103 (42.3)
≥ 24	2989 (69.3)	1181 (91.1)	302 (73.7)	1506 (57.7)
Marital status				
Single	1773 (54.7)	658 (50.7)	171 (53.9)	944 (58.1)
Non-single	1467 (45.3)	639 (49.3)	146 (46.1)	682 (41.9)
Tianjin household register				
Local	131 (3.0)	42 (3.2)	17 (4.1)	72 (2.8)
Non-local	4183 (97.0)	1255 (96.8)	393 (95.9)	2535 (97.2)
Time living in Tianjin				
≤ 2 year	1247 (41.3)	532 (41.1)	119 (40.8)	596 (41.5)
> 2 years	1776 (58.7)	762 (58.9)	173 (59.2)	841 (58.5)
Level of education				
Junior school or below	975 (30.1)	467 (36.0)	90 (28.4)	418 (25.7)
High school	1227 (37.9)	481 (37.1)	114 (36.0)	632 (38.9)
College and above	1037 (32.0)	348 (26.9)	113 (35.6)	576 (35.4)
Method of seeking sexual partners				
bathhouse	2229 (60.5)	668 (51.5)	207 (52.4)	1354 (68.0)
internet	1180 (32.0)	488 (37.6)	163 (41.3)	529 (26.5)
other	275 (7.5)	141 (10.9)	25 (6.3)	109 (5.5)
Male sex worker				
yes	216 (5.0)	35 (2.7)	12 (2.9)	169 (6.5)
no	4099 (95.0)	1262 (97.3)	398 (97.1)	2439 (93.5)
HIV infection				
yes	353 (8.2)	102 (7.9)	54 (13.2)	197 (7.6)
no	3963 (91.8)	1195 (92.1)	356 (86.8)	2412 (92.4)

### HIV infection

Among HIV infections, the prevalence of non-annual testers was the highest, reaching 13.2%, followed by never tested (7.9%) and annual testers (7.6%). (Table 1).

### Sexual behavior and HIV/AIDS related knowledge differences

In the past 6 months, 94.5% of the MSM had anal sex with men. Most of the annual testers (54.5%) always used condoms, while the first-tested (66.3%) and non-annual testers (56.9%) tended to use condoms occasionally. Overall, most MSM (80.1%) used condoms during their last anal sex. The proportion of group sexual encounters (GSEs) was highest among non-annual testers (23.9%) and lower among those who were never tested (10.7%). One thousand four hundred and sixty (1460, 33.8%) MSM had sexual intercourse with a woman in the last 6 months, and among non-annual and first-testers, the proportion was over 37%. The proportion of MSM who received the condom promotion within the last 1 year among the annual testers was 88.4%, followed by the non-annual testers (32.7%) and first-tested (17.7%). Similarly, annual testers (80.9%) had the highest level of HIV/AIDS knowledge, non-annual testers (60.2%) and first-tested MSM (48.2%) had a lower level of knowledge. (Table 2).

**Table 2:** HIV and AIDS knowledge and high-risk sexual behaviors of different testers

variable	total	First-tested	Non-annual	Annually-tested
		N (%)	N (%)	N (%)
Frequency of going to the bathhouse				
Once a month	3575 (97.0)	1272 (98.1)	390 (98.7)	1913 (96.0)
Once a week	60 (1.7)	13 (1.00)	4 (1.0)	43 (2.2)
Twice or more a week	49 (1.3)	12 (0.9)	1 (0.3)	36 (1.8)
Had anal intercourse (AI) in the last six months				
yes	4077 (94.5)	1202 (92.7)	383 (93.4)	2492 (95.5)
no	239 (5.5)	95 (7.3)	27 (6.6)	117 (4.5)
The latest AI condom use				
yes	3274 (80.1)	918 (76.0)	281 (73.2)	2075 (83.1)
no	815 (19.9)	290 (24.0)	103 (26.8)	422 (16.9)
Frequency of condom use				
never used	112 (2.7)	56 (4.7)	14 (3.7)	42 (1.7)
sometimes	2109 (51.7)	799 (66.3)	218 (56.9)	1092 (43.8)
always uesd	1860 (45.6)	350 (29.0)	151 (39.4)	1359 (54.5)
Had group sex in the past six months				
yes	650 (15.1)	139 (10.7)	98 (23.9)	413 (15.8)
no	3665 (84.9)	1157 (89.3)	312 (76.1)	2196 (84.2)
Had AI with commercial male sex partner(s)				
yes	423 (9.8)	75 (5.8)	37 (9.0)	311 (11.9)
no	3892 (90.2)	1222 (94.2)	373 (91.0)	2297 (88.1)
Had sex with any female partner(s)				
yes	1460 (33.8)	488 (37.6)	152 (37.1)	820 (31.4)
no	2856 (66.2)	809 (62.4)	258 (62.9)	1789 (68.6)
Accept condom promotion in the past year				
yes	2669 (61.9)	229 (17.7)	134 (32.7)	2306 (88.4)
no	1646 (38.1)	1068 (82.3)	276 (67.3)	302 (11.6)
HIV-related knowledge				
≤ 6	1334 (30.9)	672 (51.8)	163 (39.8)	499 (19.1)
> 6	2982 (69.1)	625 (48.2)	247 (60.2)	2110 (80.9)

### Factors related to HIV testing behavior

#### Univariate analysis results

Low levels of education (OR = 0.58, 95% CI:0.49–0.69, *p* < 0.0001) and non-bathhouse MSM (*p* < 0.05) showed annual testing was less likely. Young MSM (OR = 4.81, 95% CI: 4.13–5.61, *p* < 0.0001), single MSM (OR = 1.31, 95% CI: 1.15–1.50, *p <* 0.0001), male sex workers (MSW) (OR = 2.42, 95% CI: 1.75–3.35, *p <* 0.0001), those who received condom promotion (OR = 24.61, 95% CI: 21.07–28.74, *p* < 0.0001), visited the bathhouse frequently (OR = 2.22, 95% CI: 1.19–4.12, *p* = 0.01), had high levels of HIV/AIDS knowledge (OR = 3.95, 95% CI: 3.47–4.50, *p* < 0.0001), had group sex in the past 6 months (OR = 1.29, 95% CI: 1.09–1.54, *p* = 0.003), had anal intercourse (AI) with commercial male sex partners (OR = 1.97, 95% CI: 1.57–2.46,*p <* 0.0001), had STIs (OR = 1.58, 95% CI: 1.13–2.22, *p* = 0.008), always used condoms during anal sex in the last 6 months(*p* < 0.05) and used condoms during their last sexual encounter (OR = 1.53, 95% CI: 1.32–1.78, *p* < 0.0001) were more likely to undergo annual HIV testing. (Table 3).

**Table 3:** Univariate and Multivariate analysis on factors associated with HIV testing

Variable	Univariate		Multivariate	
	OR (95CI)	*p* value	OR (95CI)	*p* value
Age				
≤ 24	4.81 (4.13–5.61)	< 0.0001	0.67 (0.49–0.92)	0.01
> 24	1.00		1.00	
Marital status				
Single	1.31 (1.15–1.50)	< 0.0001	1.30 (1.07–1.57)	0.007
Non-single	1.00		1.00	
Place of residence				
local	1.00			
Non-local	1.20 (0.85–1.69)	0.30		
Level of education				
Junior school or below	0.58 (0.49–0.69)	< 0.0001	0.60 (0.48–0.77)	< 0.0001
High school	0.82 (0.70–0.97)	0.02	0.72 (0.58–0.90)	0.004
College and above	1.00		1.00	
Frequency of going to the bathhouse				
Once a month				
Once a week	2.15 (1.23–3.75)	0.007		
Twice or more a week	2.22 (1.19–4.12)	0.01		
Method of seeking sexual partners				
bathhouse	1.00			
internet	0.56 (0.49–0.64)	< 0.0001		
other	0.41 (0.32–0.52)	< 0.0001		
HIV-related knowledge				
≤ 6	1.00	< 0.0001	0.69 (0.57–0.83)	< 0.0001
> 6	3.95 (3.47–4.50)		1.00	
The latest AI condom use				
yes	1.53 (1.32–1.78)	< 0.0001		
no	1.00			
Had group sex in the past six months				
yes	1.29 (1.09–1.54)	0.003		
no	1.00			
Had AI with commercial male sex partner(s)				
yes	1.97 (1.57–2.46)	< 0.0001		
no	1.00			
Had Sexually transmitted diseases				
yes	1.58 (1.13–2.22)	0.008		
no	1.00			
Accept condom promotion and distribution in the past year				
yes	24.61 (21.07–28.74)	< 0.0001	14.52 (12.04–17.51)	< 0.0001
no	1.00		1.00	
Male sex worker				
yes	2.42 (1.75–3.35)	< 0.0001		
no	1.00			
Frequency of condom use during anal sex in the last six months				
never	0.23 (0.16–0.33)	< 0.0001	0.50 (0.29–0.87)	0.01
sometimes	0.39 (0.34–0.45)	< 0.0001	0.69 (0.57–0.83)	0.0001
always	1.00		1.00	
HIV infection				
yes	1.12 (0.90–1.39)	0.30		
no	1.00			

#### Multivariate analysis

The multivariate logistic analysis showed that age, education level, HIV/AIDS knowledge, marital status, acceptance of condom promotion and distribution, and frequency of condom use are associated with HIV testing behaviors.

Adolescents under the age of 24 (OR = 0.67, 95% CI: 0.49–0.92, *P* = 0.01), low-educated MSM (OR = 0.60, 95% CI: 0.48–0.77, *P* < 0.0001) and those with low levels of HIV/AIDS knowledge (OR = 0.69, 95% CI: 0.57–0.83, *P <* 0.0001) are less likely to undergo annual testing.

Those who always use condoms during anal intercourse (*p* < 0.05), received condom promotion and distribution within the last 1 year (OR = 14.52, 95% CI: 12.04–17.51, *p* < 0.0001) and single MSM (OR = 1.30, 95% CI: 1.07–1.57, *p* = 0.007) are more likely to have undergone annual testing. (Table 3).

## Discussion

Our results demonstrate that MSM are not inclined to undergo annual HIV testing. Additionally, those MSM who are young, married, less-educated, have low-level HIV/AIDS related knowledge and those who did not accept condoms use and promotion are less likely to undergo annual HIV testing [18–20]. MSM is a high-risk group for HIV infection and should be tested at least annually or more frequently due to their ongoing risky behaviors [21].

The HIV testing done shows that MSM may not undergoing HIV testing regularly as expected [22]. Their HIV testing results indicate that 29% of the HIV-infected MSM had never been tested for HIV before, 15% had not tested for HIV within the last 1 year, while the HIV infection rate among non-annual testers was 13.2%, the highest among the three groups. At least 44% of MSM with possible long-term HIV infection had not undergone regular testing.

The transmission rate of those who do not know their HIV status is estimated to be three times that of those who are aware of their status [23]. The lack of awareness of HIV testing, negative emotions such as the inconvenience of detection, perceived low service quality, and anxiety about HIV related stigma may cause MSM to avoid HIV testing for long periods [24].

The young MSM and those with low-education levels are main target groups for HIV testing [25]. The HIV infection rate among young MSM Chinese has been increasing [26]. Young MSM may have strong sexual desire, maybe more likely to contact MSM groups and have male sexual intercourse following online internet communication [27]. However, lack of HIV/AIDS knowledge and fear of positive results may discourage young MSM from undergoing HIV testing [28]. HIV/AIDS education was associated with significant additional reductions in sexually risky behaviors among Young MSM [29]. Low-educated MSM tend to test less for HIV, which is consistent with previous reports [30]. It is important to adopt different online and internet sites using new technologies to strengthen the HIV/AIDS related knowledge to improve HIV testing in young people [31]. Interventions amongst farmers, workers and other low-educated MSM can be implemented through easy-to-understand methods, such as using pictorial brochures to promote free HIV testing and safe sex education [32]. Therefore, when low-educated MSM seek sexual partners or engage in group sex events in the bathhouses, these interventions may reduce their risk of HIV infection.

Our results indicate that the annual testing behavior of married MSM is not as good as that of single MSM. It has been reported in China that due to parental or social pressure, nearly half (48.9%) of MSM chose to marry women, and 91% of them reported that they would practice homosexual behavior after marriage [33]. This behavior may expand the spread of HIV from MSM to the general population or even the next generation through HIV maternal transmission. It is therefore essential to increase the frequency of HIV testing in married MSM, such as making HIV testing more accessible and by promoting self-test kits. In addition, provision of frequent HIV testing to married MSM may contribute in promoting HIV testing among their sexual partners and in facilitating safer sexual practices.

The proportion of condom promotion and advanced HIV/AIDS related knowledge received in this study was about 60 to 70%, suggesting that continued expansion and strengthening of HIV-related advocacy in MSM may lead to improvements in HIV testing behaviour. In addition, the awareness of condom use is also related to frequent HIV testing [34]. Compared with MSM who always used condoms during anal sex for the past 6 months, people who never used or occasionally used condoms appeared to lack awareness of annual HIV testing. Possessing a high level of HIV/AIDS knowledge and receiving condom promotion and distribution can foster better HIV testing awareness, thereby promoting regular HIV testing behaviors among MSM [35]. Strategies to strengthen the promotion of condom use and safe sexual behaviors warrants further consideration in MSM in China to achieve the 2030 UNAIDS goal [36].

HIV/AIDS prevention and control agencies may be able to promote the benefits and implementation of HIV testing in places where MSM frequently find sexual partners such as bathhouses and the Internet. In addition, it is also necessary for the testing agency to ensure a friendly and non-discriminatory HIV testing environment for the testers [37]. Another strategy is to vigorously promote self-testing kits to ensure that potential HIV-infected MSM can safely undergo frequent HIV testing in their own homes [38].

### Limitations

Firstly, snowball sampling has several disadvantages such as community bias, non-random sampling, unknown sampling of the population size, anchoring and lack of researcher control over sampling method. However, snowball sampling was chosen to recruit MSM who are generally a marginalized population who are difficult to reach [39].

Secondly, self-administered questionnaires have several participant biases such as exaggeration, embarrassment, social desirability, emotional state and trying to satisfy investigators [40]. To overcome some of these shortcomings, the survey questionnaire was conducted by professionally trained MSM using MSM specific lingo to better improve trust and communication with the participants.

## Conclusions

Men who have sex with men (MSM) are a marginalized population with a high risk of HIV infection. Therefore, they need to undergo HIV testing at least once a year to prevent and control the spread of HIV, which we report may not be uniformly practiced in China. HIV/AIDS health and education interventions should accommodate the diversity of MSM with specific focus on those MSM who are young, have low education levels and are married to improve HIV testing behavior and frequency. The aim to end the AIDS epidemic by 2030 by achieving 95% diagnosed among all people living with HIV necessitates that all relevant stakeholders implement targeted publicity, HIV/AIDS health education, and promote self-test kits to encourage HIV testing among MSM in China.

## Supplementary Information

**Additional file 1.**

## Data Availability

The datasets used and analyzed during the current study are available from the corresponding author on reasonable request.

## Abbreviations

MSM: Men who have sex with men
STD: Sexually Transmitted Disease
MSW: male sex worker
UNAIDS: The Joint United Nations Programme on HIV/AIDS
NHAS: The recent National HIV/AIDS Strategy
GSEs: Group sexual encounters
AI: Anal intercourse

## References

[1] 12019-UNAIDS-data [https://www.unaids.org/sites/default/files/media_asset/2019-UNAIDS-data_en.pdf].

[2] Zhang L, Chow EP, Jing J, Zhuang X, Li X, He M (2013). HIV prevalence in China: integration of surveillance data and a systematic review. Lancet Infect Dis.

[3] Deeks SG, Overbaugh J, Phillips A, Buchbinder S (2015). HIV infection. Nat Rev Dis Primers.

[4] Hutchinson AB, Farnham PG, Sansom SL, Yaylali E, Mermin JH (2016). Cost-effectiveness of frequent HIV testing of high-risk populations in the United States. J Acquir Immune Defic Syndr.

[5] Campbell CK, Lippman SA, Moss N, Lightfoot M (2018). Strategies to increase HIV testing among MSM: a synthesis of the literature. AIDS Behav.

[6] Liu Y, Wang J, Qian HZ, Liu H, Yin L, Lu H, Zhang C, Ruan Y, Shao Y, Vermund SH (2016). Seeking male sexual partners via internet and traditional venues among Chinese men who have sex with men: implications for HIV risk reduction interventions. AIDS Behav.

[7] Myles RL, Best J, Bautista G, Wright ER, LaBoy A, Demissie Z, Dean HD (2020). Factors associated with HIV testing among Atlanta's homeless youth. AIDS Educ Prev.

[8] Krishnamoorthy Y, Rehman T, Sakthivel M (2021). Effectiveness of financial incentives in achieving UNAID fast-track 90-90-90 and 95-95-95 target of HIV care continuum: a systematic review and Meta-analysis of randomized controlled trials. AIDS Behav.

[9] HIV testing in the United States [https://www.cdc.gov/nchhstp/newsroom/docs/factsheets/hiv-testing-us-508.pdf].

[10] Jiang H, Hong H, Dong H, Jiang J, He L (2020). HIV testing and risks of sexual behavior among HIV-negative men who have sex with men in Ningbo, China. Int J Environ Res Public Health.

[11] Clark HA, Oraka E, DiNenno EA, Wesolowski LG, Chavez PR, Pitasi MA (2019). Men who have sex with men (MSM) who have not previously tested for HIV: results from the MSM testing initiative, United States (2012-2015). AIDS Behav.

[12] Croxford S, Tavoschi L, Sullivan A, Combs L, Raben D, Delpech V, Jakobsen SF, Amato-Gauci AJ, Desai S (2020). HIV testing strategies outside of health care settings in the European Union (EU)/European economic area (EEA): a systematic review to inform European Centre for Disease Prevention and Control guidance. HIV Med.

[13] Hess KL, Chavez PR, Kanny D, DiNenno E, Lansky A, Paz-Bailey G, NHBS Study Group (2015). Binge drinking and risky sexual behavior among HIV-negative and unknown HIV status men who have sex with men, 20 US cities. Drug Alcohol Depend.

[14] Furegato M, Mitchell H, Ogaz D, Woodhall S, Connor N, Hughes G, Nardone A, Mohammed H (2018). The role of frequent HIV testing in diagnosing HIV in men who have sex with men. HIV Med.

[15] Bai X, Xu J, Yang J, Yang B, Yu M, Gao Y, Dong WM, Wu Z (2014). HIV prevalence and high-risk sexual behaviours among MSM repeat and first-time testers in China: implications for HIV prevention. J Int AIDS Soc.

[16] Wei C, Ruan S, Zhao J, Yang H, Zhu Y, Raymond HF (2011). Which Chinese men who have sex with men miss out on HIV testing?. Sex Transm Infect.

[17] Cui Z, Huang H, Zhang T, Yu Z, Zhang H, Yao T, Song D, Chen Y, Peixoto E, Wang C, Wang X, Yang J, Liu Y, Li C, Ma J (2021). Low awareness of and willingness to use PrEP in the Chinese YMSM: an alert in YMSM HIV prevention. HIV Med.

[18] Li R, Pan X, Ma Q, Wang H, He L, Jiang T, Wang D, Zhang Y, Zhang X, Xia S (2016). Prevalence of prior HIV testing and associated factors among MSM in Zhejiang Province, China: a cross-sectional study. BMC Public Health.

[19] Sun S, Whiteley L, Brown LK (2020). HIV testing among Chinese men who have sex with men: the roles of HIV knowledge, online social life, and sexual identity concerns. AIDS Behav.

[20] Yan X, Su H, Zhang B, Li Y, Zhang L, Jia Z (2020). Adherence of HIV self-testing among men who have sex with men in China: longitudinal study. J Med Internet Res.

[21] Witzel TC, Melendez-Torres GJ, Hickson F, Weatherburn P (2016). HIV testing history and preferences for future tests among gay men, bisexual men and other MSM in England: results from a cross-sectional study. BMJ Open.

[22] Williams-Roberts H, Chang Y, Losina E, Freedberg KA, Walensky RP (2010). Frequent HIV testing among participants of a routine HIV testing program. Virulence..

[23] Hutchinson AB, Farnham PG, Duffy N, Wolitski RJ, Sansom SL, Dooley SW, Cleveland JC, Mermin JH (2012). Return on public health investment: CDC’s expanded HIV testing initiative. J Acquir Immune Defic Syndr.

[24] Zhang X, Wang N, Vermund SH, Zou H, Li X, Zhang F, Qian HZ (2019). Interventions to improve the HIV continuum of care in China. Current HIV/AIDS Reports.

[25] Seth P, Walker T, Figueroa A (2017). CDC-funded HIV testing, HIV positivity, and linkage to HIV medical care in non-health care settings among young men who have sex with men (YMSM) in the United States. AIDS Care.

[26] McLaughlin K. HIV infections are spiking among young gay Chinese. Science. 2017;355(6332):1359–9. 10.1126/science.355.6332.1359. 10.1126/science.355.6332.1359 28360273

[27] Sullivan PS, Zhang L, Ding X, Lu R, Feng L, Li X (2012). Predictors of HIV and syphilis among men who have sex with men in a Chinese Metropolitan City: comparison of risks among students and non-students. PLoS One.

[28] Wong NS, Tang W, Han L, Best J, Zhang Y, Huang S, Zheng H, Yang B, Wei C, Pan SW, Tucker JD (2017). MSM HIV testing following an online testing intervention in China. BMC Infect Dis.

[29] Raifman J, Beyrer C, Arrington-Sanders R (2018). HIV education and sexual risk behaviors among young men who have sex with men. LGBT Health.

[30] Henderson ER, Subramaniam DS, Chen J (2018). Rural-urban differences in human immunodeficiency virus testing among US adults. Sex Transm Dis.

[31] Wei L, Chen L, Zhang H, Yang Z, Liu S, Tan W, Xie W, Liu L, Zhao J, Cheng J (2019). Relationship between gay app use and HIV testing among men who have sex with men in Shenzhen, China: a serial cross-sectional study. BMJ Open.

[32] Madiba S, Alwano MG, Bachanas P, Block L, Roland M, Sento B (2019). Increasing knowledge of HIV status in a country with high HIV testing coverage: results from the Botswana combination prevention project. PLoS One.

[33] Wu W, Yan X, Zhang X, Goldsamt L, Chi Y, Huang D, Li X (2020). Potential HIV transmission risk among spouses: marriage intention and expected extramarital male-to-male sex among single men who have sex with men in Hunan, China. Sex Transm Infect.

[34] Alkaiyat A, Schaetti C, Liswi M, Weiss MG (2014). Condom use and HIV testing among men who have sex with men in Jordan. J Int AIDS Soc.

[35] Nelson LE, Wilton L, Agyarko-Poku T, Zhang N, Aluoch M, Thach CT (2015). The association of HIV stigma and HIV/STD knowledge with sexual risk behaviors among adolescent and adult men who have sex with men in Ghana, West Africa. Res Nurs Health.

[36] Chow EP, Wilson DP, Zhang L (2012). Patterns of condom use among men who have sex with men in China: a systematic review and meta-analysis. AIDS Behav.

[37] Zhang L, Xiao Y, Lu R, Wu G, Ding X, Qian HZ, McFarland W, Ruan Y, Vermund SH, Shao Y (2013). Predictors of HIV testing among men who have sex with men in a large Chinese city. Sex Transm Dis.

[38] Katz DA, Golden MR, Hughes JP, Farquhar C, Stekler JD (2018). HIV self-testing increases HIV testing frequency in high-risk men who have sex with men. JAIDS.

[39] Magnani R, Sabin K, Saidel T, Heckathorn D (2005). Review of sampling hard-to-reach and hidden populations for HIV surveillance. Aids..

[40] Lavrakas, Paul J. Encyclopedia of Survey Research Methods. Thousand Oaks: Sage Publications, Inc.,; 2008. 10.4135/9781412963947.n522.

