# Supplementary material for: Factors related to HIV testing frequency in MSM based on the 2011–2018 survey in Tianjin, China: a hint for risk reduction strategy
Source: BMC Public Health. 2021 Oct 20;21:1900. doi: 10.1186/s12889-021-11948-6 (PMC8527634; doi:10.1186/s12889-021-11948-6)
Supplement: Supplementary file 1 — Additional file 1. [file 12889_2021_11948_MOESM1_ESM.docx]

**Additional files: Survey Questionnaire**

**MSM Health Registration Form**

A01 Place: Tianjin

A02 Sentinel type: MSM

A03 GB code of local administrative divisions: 120000

A04 No.□□□□(0001—9999)
A05 Fill in date □□□□year □□month □□day

Hello, Tianjin Center for Disease Control and Prevention and Tianjin Shenlan Organization office have jointly established a gay bathhouse health service station in order to provide more convenient testing services. As part of the testing services, this registration form can help us understand your knowledge and behavior about some health problems. In order to provide you with better service, please fill in truthfully. Please be assured that the information is completely confidential and will not be disclosed to unrelated personnel. If you have any questions during the registration process, the staff will be ready to help you.

**Part 1 Demographic information**

B01 Year of birth ______

B02 Marital status with the female:

1. Unmarried ②In marriage ③Cohabitation ④Divorced or widowed

B03 Location of household registration:

1. Tianjin
2. Other province (Please specify: _____)
3. Foreigner (Please specify: ______)

B04 Nationality: ______

B05 How long have you lived in Tianjin?

①<3Months ②3~6Months ③7~12Months ④1 ~2 years ⑤2 Years or more

B06 Education level:

1. Illiterate
2. Primary school
3. Junior high school
4. Senior high or secondary school

⑤ College or above

B07 In the past six months, how many times have you been to this bathhouse?

①Once a month ②Once a week ③Twice or more a week

B08 What is your most important route to find a male sexual partner?

(1) Bar / Dance hall

(2) Tea house / Club

(3) Bath

(4) Park / Public toilet / Lawn

(5) Internet / Dating software

(6) Other places (please specify: )

**Part 2 HIV/AIDS Knowledge**

C01 Can you tell a person infected with HIV from the appearance?

①Yes ②No ③I don't know

C02 Can mosquito bites spread HIV?

①Yes ②No ③I don't know

C03 Can someone be infected with HIV by eating together with an HIV-infected person or AIDS patient?

①Yes ②No ③I don't know

C04 Can someone get HIV by transfusion with blood containing HIV?

①Yes ②No ③I don't know

C05 Is it possible to get HIV if share syringes with people living with HIV?

①Yes ②No ③I don't know

C06 Is it possible that a baby born by an HIV-positive women is infected with HIV?

①Yes ②No ③I don't know

C07 Can proper use of condom reduce HIV transmission?

①Yes ②No ③I don't know

C08 Can having sex with only one sexual partner reduce HIV transmission?

①Yes ②No ③I don't know

**Part 3 Sexual behaviors**

D001 The age when you first had sex with a man______

D01 In the past six months, have you ever had sex with a male?

①Yes ②No

D011 In the past six months, how many people of the same sex have you had anal sex with? ______

D02 In the past week, how many times have you had anal sex with male? ______

D021 In the past week, how many people of the same sex have you had anal sex with? ______

D03 Did you use condoms the last time you had sex with male?

①Yes ②No

D04 In the past six months, how often do you use condoms when you had anal sex with male?

①Never ②Sometimes ③always.

E01 In the past six months, have you ever had commercial sex with male?

①Yes ②No(Jump to F01)

E02 In the past six months, how often do you use condoms in commercial sex?

①Never ②Sometimes ③always

E03 Did you use condoms the last time you had commercial sex with male?

①Yes ②No

F01 In the past six months, have you ever had sex with female?

①Yes ②No(Jump to G01)

F02 In the past six months, how often do you use condoms when you had sex with female? ①Never ②Sometimes ③always

F03 Did you use condoms the last time you had sex with female?

1. Yes ②No

G01 In the past six months, have you had group sex with male (including with three persons)?

1. Yes ②No

G02 Do you use condoms when you have group sex (including with three persons)?

①Never ②Sometimes ③always

**Part 4 Drug use**

H01 Have you ever used drugs (including meth, ketamine and other new drugs)

1. Yes ②No

H02 Have you ever injected drugs?

1. Yes ②No

H03 Have you ever shared needles with others?

1. Yes ②No

H04 When injecting drugs in the past six months, how often do you share needles with others?
① Never ② Sometimes ③always

**Part 5 Sexually transmitted diseases**

I01 In the past year, have you ever been diagnosed with a sexually transmitted disease?

1. Yes ②No (Jump to J01)

I02 In the past year, what sexually transmitted disease have you been diagnosed?
①Gonorrhea ②Syphilis ③Chlamydia infection ④Condyloma acuminatum ⑤Genital herpes ⑥Other (please specify ) _______

**Part 6 Perception of HIV risk**

J01 How likely do you think you may be infected with HIV currently?

Very high Relatively high Moderate ④ Relatively low ⑤Very low or not

**Part 7 Utilization of services**

In the past 6 months, have you received the following services for HIV prevention?

K01 Condom promotion and distribution

1. Yes ②No

K02 Community medication maintenance treatment/cleaning needle supply or exchange

1. Yes ②No

L01 In the past year, have you had HIV test?

①Yes ②No (end of survey)
L02 Do you know the test results yourself?

1. Yes ②No

L03 When was your last test? (If you have never done it before, leave it blank, end)

Year _______

Month _______

L04 What was the result of your last test?

①negative ②positive ③I don't know

Registration is over, thank you!
-------------------------------------------------------------------------------------------------------------------

The following are filled out by the staff:

M01 Is the bathhouse patron an MSW？

Yes No

HIV screening test results:

T01 Whether blood was collected in this investigation.

Yes No

T02a Whether it has been tested positive for HIV in the past?

Yes No(jump to T03)

T02b When is the earliest confirmation test positive?

Year _______

Month _______

T03 HIV oral rapid test:

Reactive Non-reactive

T04 HIV blood rapid test:

Reactive Non-reactive

HIV Confirmatory test results:

T05 Have the patron received HIV confirmatory test？

Yes No

T06 HIV Confirmatory test results?

Positive Negative
